# Supplementary material for: Involvement of the Iron-Regulated Loci hts and fhuC in Biofilm Formation and Survival of Staphylococcus epidermidis within the Host
Source: Microbiol Spectr. 2022 Jan 12;10(1):e02168-21. doi: 10.1128/spectrum.02168-21 (PMC8754135; doi:10.1128/spectrum.02168-21)
Supplement: SUPPLEMENTAL FILE 1 — Supplemental material. Download SPECTRUM02168-21_Supp_1_seq8.pdf, PDF file, 2.8 MB [file spectrum02168-21_supp_1_seq8.pdf]

Supplementary information for

Involvement of the iron-regulated loci *hts* and *fhuC* in biofilm formation and survival of *Staphylococcus epidermidis* within the host

Fernando Oliveira[1,2,3], Tânia Lima[3], Alexandra Correia[3], Ana Margarida Silva[3], Cristina Soares[4], Simone Morais[4], Samira Weißelberg[2], Manuel Vilanova[3,5,6], Holger Rohde[2], Nuno Cerca[1]*

[1]Centre of Biological Engineering, LIBRO - Laboratory of Research in Biofilms Rosário Oliveira, University of Minho, Campus de Gualtar, 4710-057, Braga, Portugal

[2]Institut für Medizinische Mikrobiologie, Virologie und Hygiene, Universitätsklinikum Hamburg-Eppendorf, Martinistraße 52, 20246 Hamburg, Germany

[3]i3S - Instituto de Investigação e Inovação em Saúde, Universidade do Porto, Rua Alfredo Allen, 4200-135, Porto, Portugal

[4]REQUIMTE-LAQV, Instituto Superior de Engenharia do Porto, Instituto Politécnico do Porto, Rua Dr. António Bernardino de Almeida, 431, 4249-015 Porto, Portugal

[5]IBMC, Instituto de Biologia Molecular e Celular, Universidade do Porto, R. Alfredo Allen, 4200-135 Porto Porto, Portugal

[6]ICBAS-UP, Instituto de Ciências Biomédicas de Abel Salazar, Universidade do Porto, R. Jorge de Viterbo Ferreira 228, 4050-313, Porto, Portugal

*Corresponding author: Nuno Cerca

Supplementary Figures

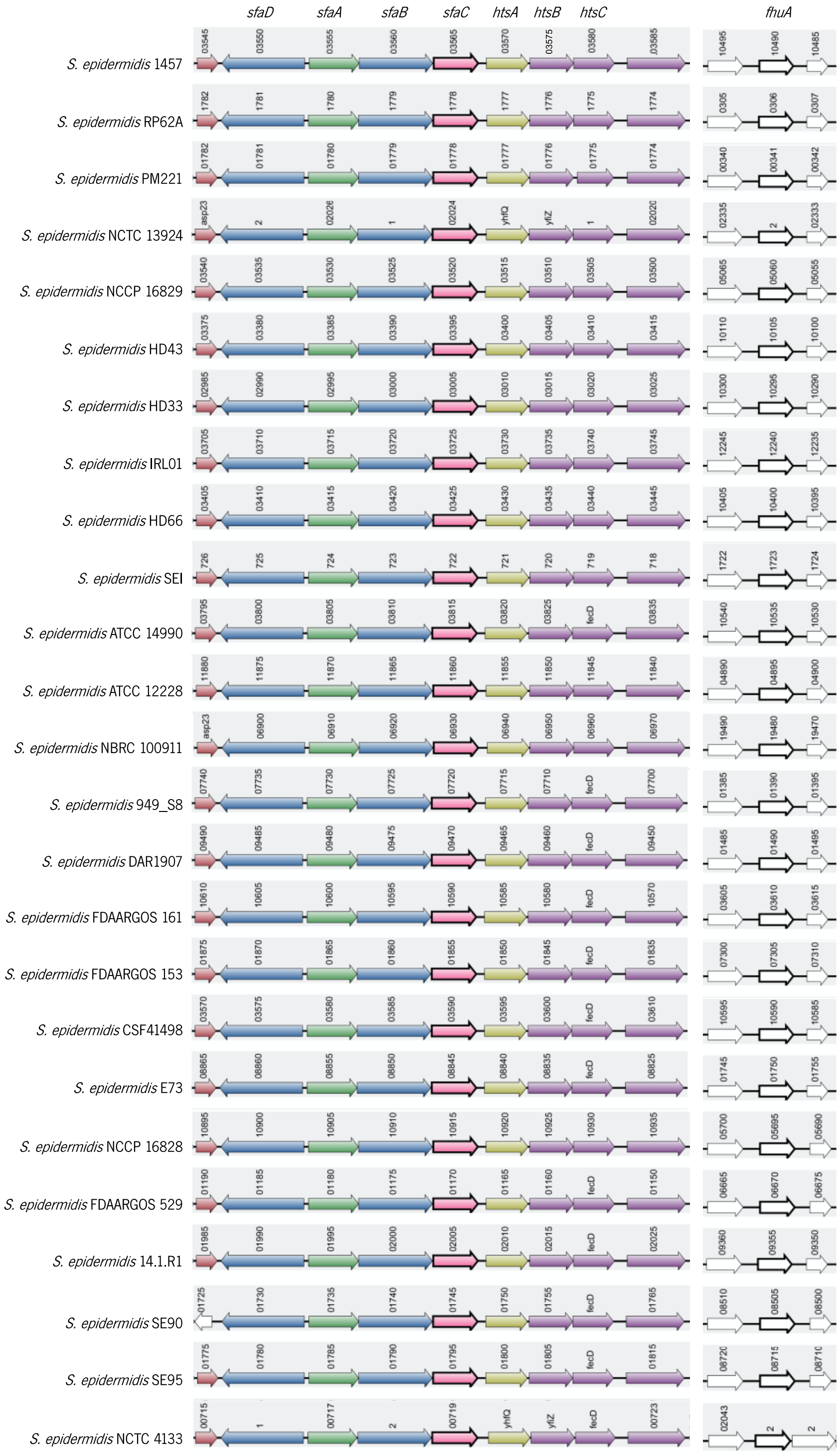

Figure S1. Genomic organization of *hts*, and *fhuC* loci in available *S. epidermidis* genomes. Open reading frames are indicated by arrows, which show the direction of transcription. Genes encoding proteins of the same family are depicted in the same color.

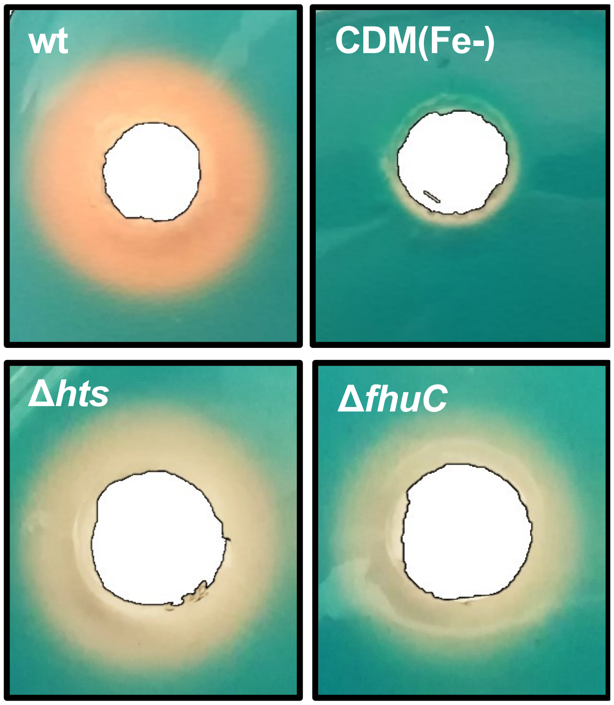

Figure S2. Deletion of *hts* and *fhuC* does not compromise siderophore production. Strains were grown in CDM(Fe-) for 72 h at 37ºC and the culture supernatants were tested for siderophore production using a modified CAS agar diffusion assay. The formation of an orange halo around each hole is indicative of siderophore presence in the supernatant.

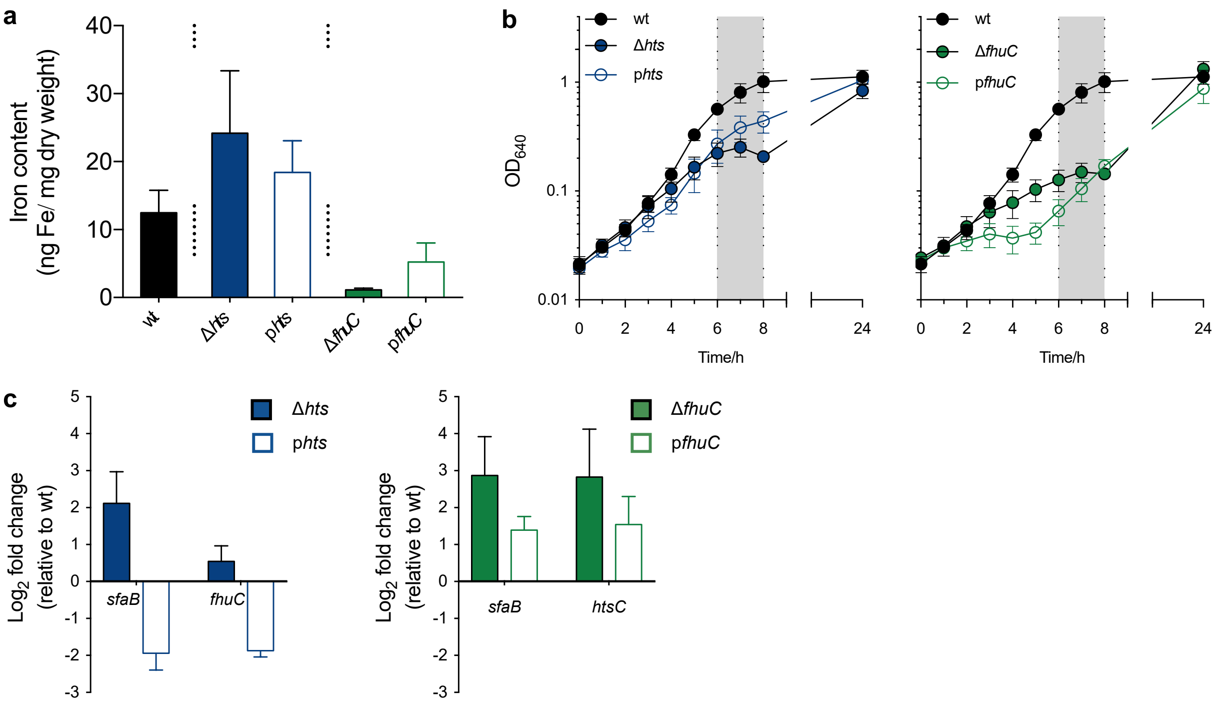

Figure S3. Expression of *hts* and *fhuC* loci *in trans* fully/partly reverses the cell iron content, planktonic growth and transcript levels of selected genes exhibited by the mutant strains. a, Analysis of the cellular iron content by atomic absorption spectroscopy (*N* = 3). b**,** Strains were allowed to grow for 24 h at 37ºC, 120 rpm in CDM(Fe-) and growth was monitored as means of OD_{640} (*N* = 3). c**,** Transcription of iron acquisition-associated genes after culture in CDM(Fe-) for 24 h. Fold change data were calculated according to Pfaffl method and log-transformed (Log_2). Values above and below 0 indicate up- and down-regulation of transcription, respectively, relative to wt (*N* = 3). The bars (**a**, **c**) and symbols (**b**) represent the mean of biological replicates and the error bars represent the s.e.m.

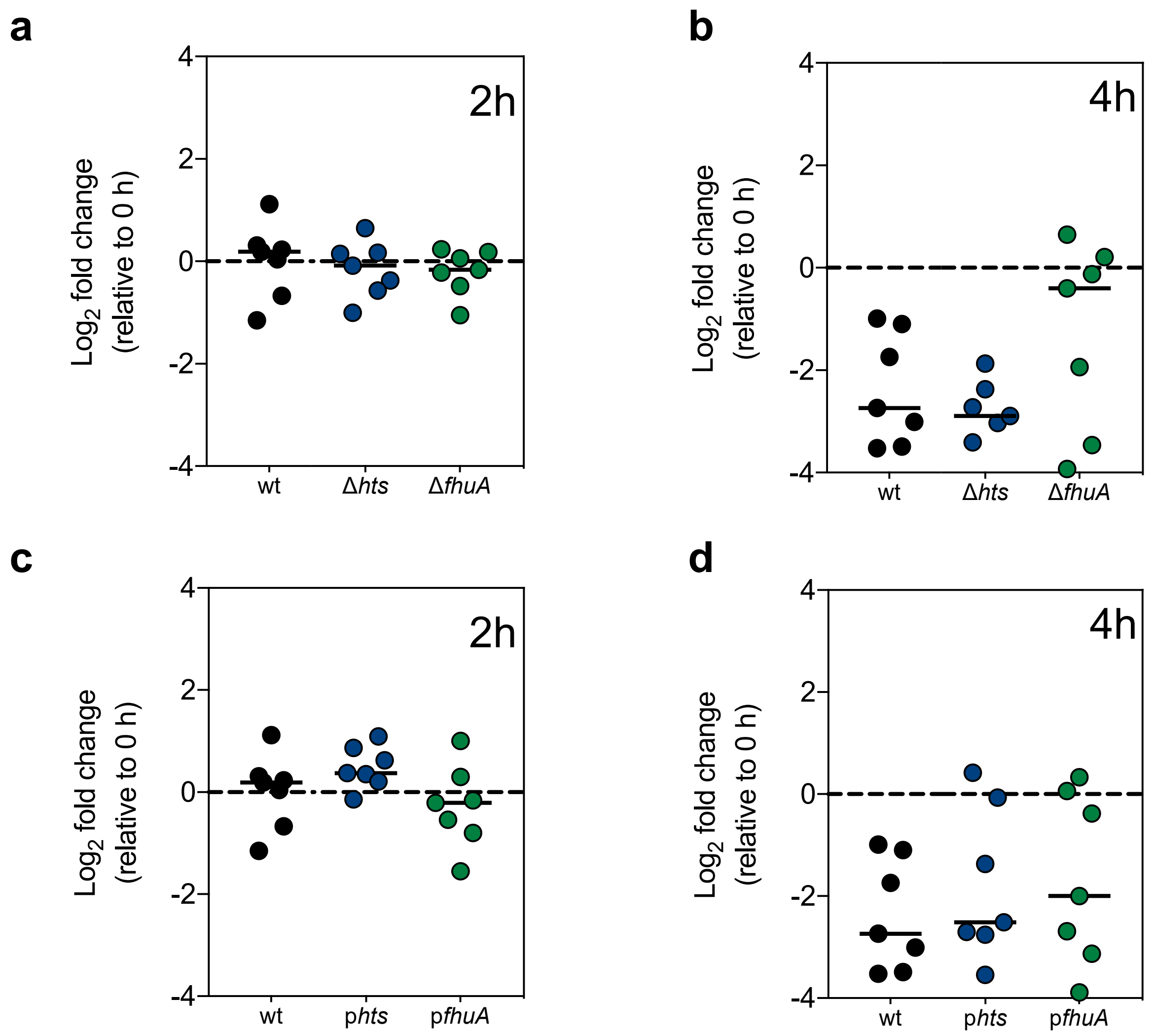

Figure S4. ***S. epidermidis*** **survival in human blood is not impacted by the lack of either** ***hts*** **or** ***fhuC*****.** *S. epidermidis* wt, mutant (**a**,**b**) and complementation strains (**c**,**d**) were incubated with human blood for the desired period and CFU were counted after 1-2 days at 37 °C. Each point represents the fold change in CFU (versus 0 h) obtained in one experiment with a single donor and lines represent the median from biological replicates (n = 7 donors). Values above and below 0 indicate bacterial replication and clearance, respectively. No significant differences were found using one-way ANOVA with Dunnett's multiple comparisons test.

Supplementary tables

Table S1. Bacterial strains, plasmids and phages used in this study

Strain, plasmid or phage	Description*	Reference
E. coli		
DH5α	Chemically competent cells for cloning purposes	New England Biolabs (NEB)
TOP10	Chemically competent cells for cloning purposes	Thermo Fisher Scientific, Inc
S. aureus		
RN4220	Derived from NCTC8325-4; r_k^- m_k^+; accepts foreign DNA	(1)
PS187 Δ*hsdR* Δ*sauPSI*	*S. aureus* PS187 strain deficient in type IV and type I restriction systems	(2)
S. epidermidis		
1457	Wyld-type clinical isolate from a central venous catheter infection; *icaADBC*$^+$, *aap*$^+$, *embp*$^+$, strong biofilm formation	(3)
1457Δ*agr*::*spcR*	Mutant carrying a deletion of the accessory gene regulator (*agr*) system; Spt^R	(4)
1457Δ*hts*::*dhfr*	Mutant carrying a deletion of *htsABC*; Tmp^R	This study
1457Δ*fhuC*::*ermC*	Mutant carrying a deletion of *fhuC;* Ery^R	This study
1457Δ*hts* p*hts*	Complemented mutant 1457Δ*hts*; *in trans* expression of *hts* from its natural promoter; Tmp^R, Cm^R	This study
1457Δ*fhuC* p*fhuC*	Complemented mutant 1457ΔfhuC; *in trans* expression of *fhuC* from its natural promoter; Ery^R, Cm^R	This study
**Plasmids**		
pBASE6	Temperature-sensitive suicide mutagenesis vector; Amp^R, Cm^R	(5)
pB-*hts*	pBASE6 derivative containing *hts*::*dhfr;* Cm^R, Tmp^R	This study
pB-*fhuC*	pBASE6 derivative containing *fhuC*::*ermC*; Cm^R, Ery^R	This study
pRB473	Shuttle vector for cloning in *E. coli* and staphylococci; constitutive gene expression in staphylococci via *vegII* promoter	(6)
p*hts*	pRB473 derivative containing *htsABC* and its natural promoter	This study
p*fhuC*	pRB473 derivative containing *fhuC* and its natural promoter	This study
**Phages**		
ϕ187	*S. aureus* phage; wt	(7)
A6C	*S. epidermidis*; wt	(8)

*Abbreviations: Amp^R, Cm^R, Ery^R, Tmp^R, resistance to ampicillin, chloramphenicol, erythromycin, spectinomycin, tetracycline and trimethoprim respectively.

Table S2. Sequences of oligonucleotides used in this study

Description	Purpose	Sequence (5' - 3')*
**Δ*htsABC***		
SERP1775_UP_Fw	Amplification of fragments for construction of plasmid pB-*hts*	cccgccctgccactcatcgcagtgcagcgg**aattc**GCAATGACAATAGCGAAC (*Eco*RI)
SERP1775_UP_Rv		tattgataatgtcatTAAATTTGATAGTTTCAATATCGTATTTTTTAG
dhfR_Fw		aaactatcaaatttaATGACATTATCAATAATTGTCG
dhfR_Rv		tatacatattaaggaCTATTTCCCTTTTCTACGC
SERP1777_DOWN_Fw		agaaaagggaaatagTCCTTAATATGTATATGCTTACATTATTAG
SERP1777_DOWN_Rv		ctgcgcgctagcccgggtaccgagctccgg**aattc**GATGATTTCAAGAGTAGAAATGAG (*Eco*RI)
SERP1774_Fw ¥	Screening of plasmid integration through upstream region (5')	CTGTTTTGCCTTCTGGTAGCTG
pBASE6-dhfR_Rv		AGTGTATTCCCAGTGGTCAGT
pBASE6-dhfR_Fw	Screening of plasmid integration through upstream region (3')	TCTTTCGACTGAGCCTTTCG
SERP1775_Rv		TCTGGCTCGGTGATACAAGG
SERP1777_Fw	Screening of plasmid integration through downstream region (5')	GCTTACGTGTTCCTACAGAAG
pBASE6_DOWN_INT_Rv		CCTCGCAGCACGATATAAAG
dhfR_Fw	Screening of plasmid integration through downstream region (3')	AAACTATCAAATTTAATGACATTATCAATAATTGTCG
SERP1778_Rv ¥		GGCGAATGTTCGTGTCAAT
pRB1775-77_Fw	Amplification of fragments for construction of plasmid p*hts*	gcggaattcgagctcggtacccggg**gatcc**CTACATCTTACGTAATAAAAATAAGAAATAAG (*Bam*HI)
pRB1775-77_Rv		gaatccaagcttgcatgcctgcagg**tcgac**TAGTTCTTATTTACCTTTAACTCAAC (*Sal*I)

**Table S2.** (continued)		
**Description**	**Purpose**	**Sequence (5' - 3')***
**Δ*fhuC***		
SERP0306_UP_Fw	Amplification of fragments for construction of plasmid pB-*fhuC*	cctgccactcatcgcagtgcagcgg**aattc**GTGGGTTATAAATATTTGAGTAAAC (*Eco*RI)
SERP0306_UP_Rv		gtctcattcaattAATAATTCCCCTACTTTCATTC
ermC_Fw		taggggaattattAATTGAATGAGACATGCTAC
ermC_Rv		taagatgttgcatGAAAACTGGTTTAAGCCG
SERP0306_DOWN_Fw		taaaccagttttcATGCAACATCTTATAAAAAAACATG
SERP0306_DOWN_Rv		cgctagcccgggtaccgagctccgg**aattc**AAGACAAGAATATAATCAAAAACTTTAC (*Eco*RI)
SERP0305_Fw ¥	Screening of plasmid integration through upstream region (5')	CTCTGCAGGTATCAATGCA
ermC_Rv		taagatgttgcatGAAAACTGGTTTAAGCCG
pBASE6_UP_INT_Fw	Screening of plasmid integration through upstream region (3')	AGCTAGAGAGTCATTACCCCAG
SERP0306_Rv		GCAACCTTCTTCTTCGTTGAGC
SERP0306_Fw	Screening of plasmid integration through downstream region (5')	GTGGTGGACAAAGACAACGC
pBASE6_DOWN_INT_Rv		CCTCGCAGCACGATATAAAG
ermC_Fw	Screening of plasmid integration through downstream region (3')	taggggaattattAATTGAATGAGACATGCTAC
SERP0308_Rv ¥		ACTGTCATGATTAATGCG
pRB0306_Fw	Amplification of fragments for construction of plasmid p*fhuC*	gtgcagcggaattcgagctcggtacccggg**gatcc**AATATCAATATATTATAAAGTTAAAAAGTTG (*Bam*HI)
pRB0306_Rv		ggtgagaatccaagcttgcatgcctgcagg**tcgac**GGCAATGTCCTCCTATTG (*Sal*HI)

¥ These oligonucleotides were used for confirmation of locus deletion

* Restriction sites are underlined and in bold

Table S3. Microwave conditions for the digestion of bacterial samples

Ramp/ min	Pressure/ Psi	Temperature/ ºC	Hold/ min
5:00	150	50	10:00
10:00	200	100	10:00
10:00	200	140	15:00

Table S4. Optimized operational parameters for the graphite furnace analysis of iron

**Step**	**Name**	**Temperature/ ºC**	**Ramp/ ºC/s**	**Hold/ s**	**Time/ s**
1	Drying	80	6	20	26.7
2	Drying	90	3	20	23.3
3	Drying	110	5	10	14.0
4	Pyrolysis	350	50	20	24.8
5	Pyrolysis	1100	300	10	12.5
6	Gas adaptation	1100	0	5	5.0
7	Atomize	2000	1500	4	4.6
8	Clean	2450	500	4	4.9

References

1. Kreiswirth BN, Löfdahl S, Betley MJ, O'reilly M, Schlievert PM, Bergdoll MS, et al. The toxic shock syndrome exotoxin structural gene is not detectably transmitted by a prophage. Nature. 1983;305(5936):709–12.
2. Winstel V, Liang C, Sanchez-Carballo P, Steglich M, Munar M, Broker BM, et al. Wall teichoic acid structure governs horizontal gene transfer between major bacterial pathogens. Nature Communications. 2013;4:2345.
3. Mack D, Siemssen N, Laufs R. Parallel induction by glucose of adherence and a polysaccharide antigen specific for plastic-adherent *Staphylococcus epidermidis*: Evidence for functional relation to intercellular adhesion. Infection and Immunity. 1992;60(5):2048–57.
4. Vuong C, Gerke C, Somerville GA, Fischer ER, Otto M. Quorum-Sensing Control of Biofilm Factors in *Staphylococcus epidermidis*. The Journal of Infectious Diseases. 2003;188(5):706–18.
5. Geiger T, Francois P, Liebeke M, Fraunholz M, Goerke C, Krismer B, et al. The Stringent Response of *Staphylococcus aureus* and Its Impact on Survival after Phagocytosis through the Induction of Intracellular PSMs Expression. PLoS Pathogens. 2012;8(11):e1003016.
6. Brückner R. A series of shuttle vectors for *Bacillus subtilis* and *Escherichia coli*. Gene. 1992;122(1):187–92.
7. Pantůček R, Doškař J, Růžičková V, Kašpárek P, Oráčová E, Kvardová V, et al. Identification of bacteriophage types and their carriage in *Staphylococcus aureus*. Archives of Virology. 2004;149(9):1689–703.
8. Rohde H, Burdelski C, Bartscht K, Hussain M, Buck F, Horstkotte MA, et al. Induction of *Staphylococcus epidermidis* biofilm formation via proteolytic processing of the accumulation-associated protein by staphylococcal and host proteases. Molecular Microbiology. 2005;55(6):1883–95.
